# The Dentist: His Relations to His Patients and to His Brother Practitioners

**Published:** 1895-05

**Authors:** C. S. Lane

**Affiliations:** Oakland, Cal.


THE
AMERICAN JOURNAL
—OF—
DENTAL SCIENCE.

VOL. XXIX. BALTIMORE, MAY, 1895. NO. I.

ARTICLE I.

### THE DENTIST: HIS RELATIONS TO HIS PATIENTS AND TO HIS BROTHER PRACTITIONERS.

BY DR. C. S. LANE, OAKLAND, CAL.

Abstract of paper read before Oakland Dental Club.

I have always been inclined to think that generally a lawyer in his zeal for his client, or to carry a point in law, forgets for the time being, at least, that he is both a *man* and a *citizen*, and that he owes something higher both to himself and to his neighbor.

I hold that it is greater and more noble to be a *man* than a genius; the more of a man the higher the possibilty of his professional genius.

Then it follows that the better a man is the better a dentist he may be. The essentials are honesty, integrity, industry, carefulness and cleanliness. His family name, his social distinctions, his early educational opportunities all may help him, but none will take the place of honest, earnest effort, every day, everywhere and in every particular.

It does not follow that a man in order to be a good dentist should neglect his duty to his country or the political or social life around him, and especially to his own family. He may discharge all these duties and obligations well and faithfully and be all the more a conscientious dentist for having done so. Generous, honest-hearted, earnest men are the best anywhere; the dentist is no exception. It does not follow that a dentist should always be solemn and severe, for the longer we live the more we should admire the pleasant and cheerful side of life. Men work longer in life's work and are more agreeable to their patrons, their families and themselves by maintaining a cheerful heart and a bright eye.

The dentist in this age of the world must be a student of whatever pertains directly to his calling, whether it be the text-books or the journals giving the thoughts and modes of others, whether in college chairs or in the busy ranks of practice; and he cannot without loss to himself neglect the association with others of his work, both professionally and socially. The dentist should know himself; he should set a high standard, physically, scientifically and morally, and strive to live up to it. His office and instruments, his person and his morals must be clean.

I owe it to myself and my patrons that my rooms should be attractive, neat and well-ventilated; that my instruments shall be clean—and that means not only presentable, but sterilized, so as to be perfectly safe to use in any other mouth; and that my presence be agreeable; that no wretchedly foul catarrhal odor from the breath shall be thrown into the face of the patient; in fact, I believe if a dentist has a nasal or pharyngeal catarrh which cannot be so far ameliorated that his breath shall not be tainted thereby, he should abandon his chair-work. The body should be well bathed, the nails and hands kept perfectly clean, and no hint of tobacco or whiskey should be found on the breath or clothing.

Dentists too frequently acquire indolent habits; office life is dull and prosy. Better shorten hours of business and take air and exercise for the recuperation of bodily and mental vigor. Interest yourself in something outside your office routine, that will not altogether divert attention and effort from the life-work, but which will more fully fit the body and mind to successfully cope with the duties of your profession.

The same law that requires us to be true to ourselves requires us to be true to our patients, as worth and nobility of character always react, enriching others as well as the possessor; hence, if we are what we ought to be, and what we may be *in ourselves*, we are so much the more to our patrons. For it cannot be too strongly emphasized that we must first of all have our own respect and consciousness of worth if we would win the respect and confidence of our patients. We may not be brilliant or win glory of men, but our own respect is worth more to us than all the flattering eulogies that men can receive who at the same time in their own breast have a conviction that they are hollow, craven and dishonest.

The dentist is a servant. The term may be distasteful to some; but he who is above service for his fellow-man is below the stature of manhood; to serve our race well is the highest honor to be coveted by mankind. Therefore in our dealings with our patients, let our first idea be that of the highest and best service; and here we are master as well as servant, for we should not only serve, but we should know what kind of service will best meet the case, and be prepared to render that service regardless of the ignorant or mistaken notions of our patients. I am only sorry for the man who will sacrifice a valuable organ to gratify an ignorant and impulsive sufferer for fear he will let slip a paltry dollar, which may possibly drop into the pocket of some unscrupulous knight of the forceps. Better a thousand times say "No!"

The struggle for gain enters so largely into all our work that many, if not all, of the nobler, more kindly virtues are suppressed as only fit to be exercised by philanthropists or religious cranks. The professions have tried by their ethical teachings to sterilize their ranks of much of its foulness, but the imagined needs of man are such that to possess them he turns a deaf ear to fraternal feeling.

An important question in our fees is: Shall we have a uniform fee for our time or for the operations involving ordinarily a certain amount of time and skill?

The dentist is often called upon to serve without remuneration; when this is the case, he should render that service with all tenderness and thoroughness, and take his reward in the consciousness of having benefited a fellow-being. However, the cheap-John quacking individual who fixes a twenty-five or fifty cent rate for extracting, and a dollar for fillings, and perhaps nothing at all for treating, with plates from five to ten dollars each, is simply degrading a noble profession. There are many people who are indiscriminating and simple enough to think that a filling is a filling, whether it costs one dollar or ten dollars. So long as they are relieved of present suffering, they make little account for the future consequences.

Not many weeks ago a patient came to me with neuralgia, which had continuously destroyed her rest for nearly three weeks previously. She had all this time been under the care of one of those kind of dentists, had a number of fillings inserted, such as they were, and one good tooth extracted from the left side of the mouth, hoping thereby to relieve the pain in the nerves of the face on the right side. How is that for science? I found an exposed and badly inflamed pulp in a bicuspid on the right side, each side of which the operator had inserted fillings, carefully avoiding this tooth, telling the patient it was a dead tooth. I saved the tooth and made it useful, but sacrificed the pulp.

Now, I maintain, such conduct and such dentists fail so far of coming up to the standard in themselves and towards their patients and the profession that they are simply parasites which we should brush off if we possibly can. The general good demands that such disgraceful conduct should be punished.

It is a notable fact that every cause and enterprise of the present day can only prosper and be successful by union and consolidation; single-handed enterprises are, as a rule, a failure. As I understand it, we are now building up a distinct profession which is young, vigorous and progressive, though not yet perfectly understood and appreciated by the public. We are bound to organize and unite our sympathies and energies to uphold and advance dental science, even if it should cost us a little personal sacrifice.—*Pacific Coast Dentist.*

---

ARTICLE II.

